# Assessment of the Prevalence of Pulp Stones in a Sample of Turkish Central Anatolian Population

**DOI:** 10.1100/2012/804278

**Published:** 2012-05-03

**Authors:** Hakan Çolak, Ahmet Arif Çelebi, M. Mustafa Hamidi, Yusuf Bayraktar, Tuğba Çolak, Recep Uzgur

**Affiliations:** ^1^Department of Restorative Dentistry, Kırıkkale University Faculty of Dentistry, 71100 Kırıkkale, Turkey; ^2^Department of Orthodontics Dentistry, Kırıkkale University Faculty of Dentistry, 71100 Kırıkkale, Turkey; ^3^Department of Prosthodontics, Kırıkkale University Faculty of Dentistry, 71100 Kırıkkale, Turkey

## Abstract

*Objective*. The aim of this study was to determine the prevalence of pulp stones (PS) in a Turkish dental patient population with respect to sexes and dental localization in relation between sex and this anomaly. *Materials Methods*. A retrospective study was performed using bitewing radiographs of 814 patients ranging in age from 15 to 65. All data (age, sex, and location) was obtained from the files. These patients were analyzed for pulp stones. Descriptive characteristics of sexes, jaws, and dental localization were recorded. The Pearson chi-squared test was used. *Results*. Of the patients, 462 (56.8%) were female and 352 (43.2%) were male. Sixty (12%) had one or more teeth that contained pulp stones. Pulp stones were identified in 518 (63.6%) of the subjects and in 2391 (27.8%) of the teeth examined. Pulp stone occurrence was significantly more common in the females than in males. With the increasing of age, the prevalence of pulp stones increased. Molars had statistically more pulp stones than premolars. Pulp stones were significantly more common in the maxilla compared with mandible. *Conclusion*. Prevalence of pulp stones in Turkish population was 27.8% but further larger-scale studies are required to assess its prevalence in the general population to compare it with other ethnic groups.

## 1. Introduction

Pulp stones (PSs) are calcified bodies in the dental pulps of the teeth in the primary and permanent dentition. They can be seen in the pulps of healthy, diseased, and even unerupted teeth [1]. Pulp stones may be located in the coronal or radicular pulp, where they may be free, attached, or embedded in the dentine. They may range in size from a macroscopic to microscopic mass, less than 200 *μ*m, beyond radiographic resolution [2]. Pulp stones were histologically classified by Kronfeld and Boyle [3] into “true” or “false” forms, the former containing irregular dentine and the latter being degenerative pulp calcifications. Other studies have noted problems with the above classification and new histological classifications have been proposed [4–6].

Some factors that have been implicated in pulp stone formation include age [7, 8], impaired pulpal blood supply [9], genetic predisposition [10], or long-standing irritants such as caries, deep fillings, or abrasion [5, 9]. Pulp obliteration is most often caused by trauma [11], but it has also been described after orthodontic treatment [12, 13] or transplantation [14, 15]. In a generalized form, it is possibly a part of the aging process and is usually seen in older individuals [16]. However, generalized pulp obliteration has also been observed in certain systemic or genetic diseases [10, 17].

The frequency of occurrence of pulp stones has been reported to increase with age [8, 18]. Some studies did not find any difference in occurrence between genders [9, 18–20], whereas other studies have found females to have more pulp stones than males [19, 21, 22].

The prevalence of PS varies from 8–90%, depending on the study type, design, and radiographic technique employed [2]. Histological method of evaluation is reported to yield higher values than radiographic method [20].

The purposes of this study were to describe the prevalence of pulp stones in a sample of Turkish dental patients using bite-wing radiographs and to explore possible associations between pulp stones and sex, tooth type, dental arch, side, and dental status; to compare the results with published data.

## 2. Materials and Methods

The study design was based on that previously published studies with small modification [18, 20, 22, 23]. We designed a descriptive study composed of bite-wing radiography of 3152 patients who presented to our Restorative Dentistry, Oral Diagnosis, and Radiology Services of Dentistry Faculty, Kırıkkale University, in the city of Kırıkkale, located in the central part of Turkey between May 2009 and November 2011. All data (age and sex) was obtained from the files.

Exclusion criteria included patients who were less than 15 years of age, records with poor quality radiographs; record with radiographs of only primary teeth and patients' data those with crown, bridge, and deep restoration. Patients whose bitewing radiographs were taken bilaterally during routine radiographic examination were included in the present study. The final sample included 841 patients (352 males, 462 females, with age range of 15–65 years).

**Figure 1:**
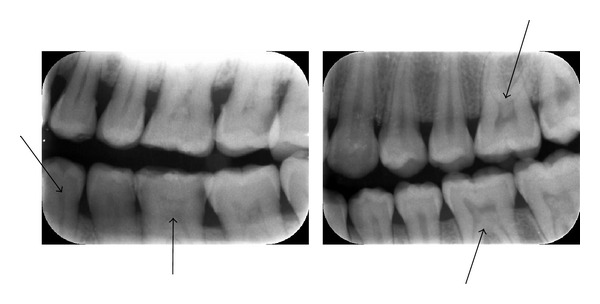
Pulp stone observed inside the pulp chambers of the molars and premolars in the bitewing radiograph.

Only the maxillary and mandibular molars (third molars were excluded) and premolars were included. Subjects with crowns or bridges that prevented adequate vision of the pulp chamber were not included in the study sample. Considering that teeth with deep fillings and caries lesions are more inclined to have pulp stones, only teeth which were noncarious and unrestored, or those with shallow fillings, were included. The radiographs were interpreted by two examiners. A tooth was recorded as having a pulp stone only when a definitive radiopaque mass was identified in the pulp chamber (Figure 1).

The reviewed radiographs were evaluated again by the same investigators one week later so that the differences between investigators could be determined. Different results were not obtained following the second evaluation. Statistical analysis of the data was performed using the SPSS computer program (SPSS 16.0, New York, USA), and the frequency distribution for pulp stones was calculated. The Pearson chi-square test was used to compare the frequency of pulp stones between male and female patients (*P* < 0.05).

## 3. Results

Bitewing radiographs of 814 patients, 352 males, 462 females, with age range of 15–65 years and average age 30.2 ± 22.4 years were studied. The bitewing radiographs of 518 patients, 206 male and 312 females, had pulp chamber calcifications. The distribution of patients having pulp stones according age groups is shown in Table 1.

Pulp stones were observed in 2391 (27.8%) of the 12928 teeth examined, 1483 in those of females and 893 in those of males. One hundred forty four patients (17.7%) had only one tooth with a pulp chamber calcification, while in 374 patients (72.2%) more than one tooth was affected. In addition, in the bitewing radiograph of one male patient, 16 teeth were detected with pulp chamber calcification. Pulp stones were detected in 1498 of the 7597 teeth (19.72%) examined in females and in 893 of the 5331 teeth (16.75%) examined in males with significant difference between the genders (*P* < 0.001, Table 2).

The distribution of pulp stones among different teeth in the upper and lower arches is shown Table 3. Pulp stones were significantly more common in the maxilla compared with mandible. Pulp stones were found in only 229 (3.74%) of the 6124 premolars and in 2162 (31.78%) of the 6804 molars examined, with differences in occurrence being statistically significant (*P* < 0.001). The frequency of pulp stones was higher in the first molars than in the second molars in each dental arch and when data for both arches were combined (*P* < 0.001, Table 4). However, in maxilla second premolars more occurred than first premolars whereas a in mandible first premolars accounted more than in second premolars. There were no statistically significant differences between the right and the left side in each tooth type and arch.

## 4. Discussion

Pulp stones are calcifications that are found in the pulp chamber or pulp canals of teeth. Structurally, pulp stones can be classified as true or false, the former being made of dentine and lined by odontoblasts, whereas false pulp stones are formed from degenerating cells of the pulp that gets mineralized [24].

Review of the literature reveals a wide discrepancy in the prevalence of pulp stones in different populations. This difference results from the variation in sample and sample size in previous studies. Furthermore, the presentations of prevalence were also different in the literature. Some investigations presented the prevalence based on person and teeth numbers [22, 23], and the others reported only the prevalence based on teeth number [18, 25]. The results of the present study on a group of Turkish dental patients have shown an overall prevalence of 63.6% for individuals and 18.5% for all teeth examined teeth. This figure is higher than the results of the study by Ranjitker et al. [20] (10.3) young Australian adults and Baghdady et al. [25] (14.8) among teenage Iraqi group and less than the study by Hamasha et al. among Jordanians (22.4%). These variations in prevalence between different populations may be due to ethnic variations and geographical differences. A recent study performed in Turkish population revealed the prevalence of pulp stones 15% [22] and 5% [23], respectively, which were lower than our findings. These contradictory findings in the same population may be explained with marked differences in the sample size.

According to the present results, there were no significant differences between left and right side occurrence (*P* > 0.05). This finding is similar to recent reports on a Turkish population [22] and Australians [20]. However, previously published studies [18, 20, 23, 25] not highlighted to pulp stones right or left side occurrence.

The prevalence of pulp stones in our sample was more frequently encountered in females than in males with significant differences between the genders in each tooth type and arch. This finding is similar to recent reports on a Iraq teenagers [25] and Turkish population [22, 26]. However, some investigators have reported that pulp stones were more common in males than in females, whereas there are also studies showing no significant differences between both sexes [20, 23]. These contradictory findings may be explained by marked differences in the sample size and in the methods used. In the literature, bruxism which causes longstanding irritation on dentition was thought to be the reason of this difference because it is more prevalent in women [27]. The statement that the effect of bruxism increases the prevalence of pulp calcifications in women is being investigated in further studies [22].

Our finding of a higher prevalence of pulp stones in the maxillary posterior teeth, especially the first molar teeth, is consistent with that of Sisman et al. [22], Tamse et al. [21], and Ranjitkar et al. [20]. In contrast, Hamasha and Darwazeh [18] found pulp stones to be more frequent in the mandibular first molar teeth.

The report of most authors [18, 20–23] supports the present one that found a predilection of pulp stones in premolars and molars in ascending order. The reason for this is unclear, but Ranjitkar et al. [20] alluded that molars, being the largest in the arch, may have a better blood supply to the pulp tissues, which may not be conducive for precipitation of more calcifications forming factors.

The structure of the normal pulp varies with advancing age. This usually leads to a progressive decrease in the number of pulp cells as well as a gradual increase in the amount of connective tissue [28]. In the literature, it was reported that subjects older than age 60 years had significantly higher prevalence of pulp stones in compared to younger age groups [21, 29]. The current finding of association between advancing age and increasing rate of PS occurrence agrees with earlier reports [6] but not with that of Hamasha et al. [18]. The increased secondary and tertiary dentine depositions, seen with advancing age, may account for this. Also, it may be a reflection of pulp's ageing process, which results in reduction in the number of fibroblasts, odontoblasts, and mesenchymal cells, which have been reported to reduce by 50% from 20–70 years [30], or presence of pulp fibrous atrophy [31]. In addition, fat deposition in the pulp may occur with age. It is reported that calcification commonly takes place within these deposits [32].

Although many studies have been carried out to explore the prevalence of pulp stones, they have differed methodology, and many prevalence studies have identified pulp stones using radiographic criteria. The true prevalence is likely to be higher than figures from these studies, because pulp stones with a diameter smaller than 200 *μ*m cannot be seen in radiographs [6]. Furthermore, in histological observations, the limited number of sections through each tooth may result in underreporting [24, 33]. In the present study, bite-wing radiographs were used. Some previous studies have used both of periapical and bite-wing radiographs [18] while someones used panoramic radiographs to identify pulp stones [27, 34]. Tamse et al. [21] examined both periapical and bitewing radiographs to identify pulp stones and to compare the two radiographic techniques and concluded that no significant difference was found between the projections.

The currently held clinical view is that pulp stones have no significance other than possibly causing difficulties during endodontic therapy, such as hindering canal location and negotiation [35]. In forensic dentistry, radiographic matching of pulp stone configurations, along with other features recorded in dental records, may provide valuable information in the identification of deceased persons [20].

Finally, due to the relatively small size sample, the prevalence figures for pulp stones in the present study should be considered with caution as they may not be a representative for the overall Turkish population. None-the-less the findings form a basis for further studies.

## Figures and Tables

**Table 1:** Distribution pulp stone (PS) by age.

Patient Age (years)	Number of patients	Number of patients with PS	% patients with PS
15–19	119	39	32.77
20–29	166	96	57.83
30–39	202	154	76.24
40–49	247	179	72.47
50–65	80	46	57.50

Total	814	514	63.14

**Table 2:** The distribution of pulp stone according to dental arches, sex, and location.

		Female						Male						Total	
		Right	%	Left	%	Total	%	Right	%	Left	%	Total	%	Total	%
	First premolar	14	0.59	11	0.46	25	1.05	9	0.38	8	0.33	17	0.71	42	1.76
Maxilla	Second premolar	17	0.71	15	0.63	32	1.34	11	0.46	8	0.33	19	0.79	51	2.13
	First molar	231	9.66	258	10.79	489	20.45	176	7.36	87	3.64	263	11	752	31.45
	Second molar	134	5.60	191	7.99	325	13.59	123	5.14	52	2.17	175	7.32	500	20.91

Mandible	First premolar	18	0.75	43	1.80	61	2.55	7	0.29	6	0.25	13	0.54	74	3.09
	Second premolar	21	0.88	27	1.13	48	2.01	7	0.29	7	0.29	14	0.59	62	2.59
	First molar	98	4.10	203	8.49	301	12.59	142	5.94	107	4.48	249	10.41	550	23
	Second molar	61	2.55	156	6.52	217	9.08	78	3.26	65	2.72	143	5.98	360	15.06

	Total	594	24.84	904	37.81	1498	62.65	553	23.13	340	14.22	893	37.35	2391	100

**Table 3:** The distribution of pulp stone according to dental arches and location.

		Right	%	Left	%	Total	%
Maxilla	First premolar	23	0.96	19	0.79	42	1.76
	Second premolar	28	1.17	23	0.96	51	2.13
	First molar	407	17.02	345	14.43	752	31.45
	Second molar	257	10.75	243	10.16	500	20.91

Mandbile	First premolar	25	1.05	49	2.05	74	3.09
	Second premolar	28	1.17	34	1.42	62	2.59
	First molar	240	10.04	310	12.97	550	23.00
	Second molar	139	5.81	221	9.24	360	15.06

Total		1147	47.97	1244	52.03	2391	100

**Table 4:** The occurrence of pulp stones in each tooth type, arch, and location.

	Maxilla								Mandible								
	Right				Left				Right				Left				Total
	FM	SM	FP	SP	FM	SM	FP	SP	FM	SM	FP	SP	FM	SM	FP	SP	
*n* (%)	407 (17.02)	257 (10.75)	23 (0.96)	28 (1.17)	345 (14.43)	243 (10.16)	19 (0.79)	23 (0.96)	240 (10.04)	139 (5.81)	25 (1.05)	28 (1.17)	310 (12.97)	221 (9.24)	49 (2.05)	34 (1.42)	2391 (100)

*n* (%)	664 (27.77)		51 (2.13)		588 (24.59)		42 (1.75)		379 (15.85)		53 (2.22)		531 (22.21)		83 (3.47)		2391 (100)

*n* (%)	715 (29.91)				630 (26.34)				432 (18.07)				614 (25.68)				2391 (100)

*n* (%)	1345 (56.25)								1046 (43.75)								2391 (100)

FM: First molar.

SM: Second molar.

FP: First premolar.

SP: Second premolar.
